# The association between caregiver well-being and care provided to persons with Alzheimer’s disease and related disorders

**DOI:** 10.1186/s13104-016-2150-z

**Published:** 2016-07-18

**Authors:** Afeez Abiola Hazzan, Harry Shannon, Jenny Ploeg, Parminder Raina, Laura N. Gitlin, Mark Oremus

**Affiliations:** Department of Medicine, McMaster University, 1280 Main Street West, Hamilton, ON L8S 4K1 Canada; Department of Clinical Epidemiology and Biostatistics, McMaster University, 1280 Main Street West, Hamilton, ON L8S 4K1 Canada; School of Nursing, McMaster University, 1280 Main Street West, Hamilton, ON L8S 4K1 Canada; Department of Community-Public Health, Center for Innovative Care in Aging, Johns Hopkins School of Nursing, Baltimore, MD 21205 USA; School of Public Health and Health Sciences, University of Waterloo, 200 University Avenue West, Waterloo, ON N2L 3G1 Canada

**Keywords:** Well-being, Quality-of-life, Quality of care, Level of care, Alzheimer’s disease and related disorders, Dementia, Caregiving, Aging

## Abstract

**Background:**

Alzheimer’s disease and related disorders (ADRD) are some of the leading causes of morbidity in developed nations. Unpaid family caregivers are primarily responsible for providing the care and support needed by persons with ADRD. In the process of caring for their loved ones with ADRD, caregivers often have to deal with multiple challenges, including their own deteriorating well-being and overall quality-of-life (QoL). A recent systematic review showed that very little research has been undertaken to study the relationship between AD caregiver QoL and the level or quality of care that caregivers provide to their loved ones. In this study, we investigate the relationships between caregiver well-being and the care provided to persons with ADRD.

**Methods:**

We used 12-month follow-up data from the Philadelphia site (n = 125) of the National Institutes of Health (NIH) multi-site study, Resources for Enhancing Alzheimer’s Caregiver Health (REACH I) to examine the relationship between caregiver well-being and the level or quality of care provided while adjusting for important covariates (e.g., age, income, and years since caregiving). Caregivers who participated in REACH I had to be at least 21 years of age and they had to be providing at least 4 h of care per day for 6 months or more to a live-in loved one with ADRD. Linear regression analysis was used to examine the relationships between well-being and the level or quality of care provided to persons with ADRD.

**Results:**

Of the 255 caregivers who participated in the REACH I study, 125 (49.0 %) remained after 12 months of follow-up. Comparisons of participants at the 12-month follow-up and participants who were lost to follow-up showed that these two sets of participants were not statistically significantly different on any of the variables examined in this study. Linear regression analysis showed that there was no statistically significant association between caregiver well-being and level or quality of care provided.

**Conclusions:**

Further research is required to investigate the factors associated with level and quality of care provided to persons with ADRD, and whether caregiver well-being (or QoL in general) is a contributor.

## Background

Alzheimer’s disease and related disorders (ADRD) are incurable conditions that reduce brain function over time. ADRD are some of the leading causes of morbidity in North America, especially among people aged 65 years or older [1]. More than 5.3 million Americans are currently living with ADRD [1, 2]. Further, one in eight older Americans currently has ADRD and up to 16 million Americans are projected to have the disease by 2050 [3, 4].

The situation in Canada is similar. Out of a population of approximately 36 million people, more than 750,000 Canadians are currently living with ADRD [5]. More than 40,000 Canadians develop these diseases annually and projections suggest that the total number of Canadians with ADRD could double to 1.4 million people by 2030 [5].

The impact of ADRD is global. A recent systematic review and meta-analysis estimated the age-standardized prevalence of ADRD in persons aged ≥60 to be 5–7 % in most world regions [2]. The authors found the highest prevalence in Latin America (8.5 %) and the lowest in sub-Saharan Africa (2–4 %) [2]. The authors also estimated that about 40 million people worldwide are currently living with ADRD, with these numbers expected to double every 20 years [2].

The majority of persons diagnosed with ADRD receive their care in the community instead of in long-term care or other assisted living facilities [6]. Among community-dwelling persons with ADRD, 80 % of their care is delivered by family caregivers [3, 5], who bear the burden of this care without receiving financial compensation [5, 7, 8]. These caregivers are usually the spouses or children of the person with ADRD.

As ADRD progresses, caregivers often have to manage increasing complexity of multiple care challenges, including changes to their own well-being [9]. Well-being is an important component of quality-of-life (QoL) and studies have shown that the QoL experienced by family caregivers of persons with ADRD is generally lower than that of caregivers who are caring for people with other chronic diseases such as cancer or acquired immune deficiency syndrome [10]. The link between caregiver well-being and the level or quality of care is important to investigate because caregivers are the primary carers for persons with ADRD. Indeed, caregivers have been called “hidden victims” because of the high social, emotional, and financial costs associated with caring for someone with ADRD [11]. However, it is not clear how the decline in ADRD caregiver well-being is related to the level or quality of care that these caregivers provide. No conceptual framework exists to specifically explain the link between caregiver QoL and the level or quality of care provided in AD. However, research evidence does link lower QoL to greater absences from work and reduced job productivity [12]. A nationally representative survey in the United States showed that lower QoL among working adults increased absences from work and reduced job productivity [12]. In the domain of care provision, these workplace productivity issues might translate into declining ‘caregiver productivity,’ which is conceived as the level and quality of care that caregivers provide to persons with AD. The study of factors that affect whether caregivers deliver optimal care is necessary to promote favorable outcomes among care recipients and also to design effective interventions that support both the quality of life of caregivers and persons with dementia.

A recent systematic review found very little published information about the relationship between AD caregiver QoL and the level or quality of care provided [13]. The systematic review included only one study, by Gitlin et al. [14], that recruited ADRD caregivers from the Philadelphia site of the Resources for Enhancing Alzheimer’s Caregiver Health (REACH I) research project. Although Gitlin et al. collected data on caregiver well-being (an important component of QoL [13]), level of care provided, mastery, and skill enhancement, the purpose of their research was to examine the six-month effects of a Home Environmental Skill-Building Program (ESP) on caregiver well-being and care recipient functioning. Consequently, Gitlin et al.’s study was not focused on the association between caregiver well-being and the level or quality of care. The strength of evidence using GRADE was “moderate,” thereby indicating that further research would be necessary to examine whether caregiver well-being and the level or quality of care are related [15, 16].

In their site-specific study as part of the National Institutes of Health (NIH) REACH I initiative [14], caregiver overall well-being was measured with a 13-item scale [the perceived change index (PCI)]. The relevant level of care measure was caregiver time (the *amount of time* devoted to providing care and total hours of instrumental activities of daily living or IADL help). The quality of care measures included caregiver mastery and skill enhancement. Caregiver mastery was measured with the caregiving mastery index (CMI). The CMI is a six-item scale evaluating the caregiver’s appraisal of his or her ability to provide care to the care recipient (CR) (e.g., “How often do you feel you should be doing more for care recipient?). Skill enhancement was measured with the task management strategy index (TMSI), which is a 19-item scale that measures the extent to which positive caregiving strategies were used to manage activities of daily living (ADL) dependence and problem behaviours in care recipients.

Since the REACH I data used in the Gitlin et al. study [14] contained information that could help to directly assess the relation between well-being and level or quality of care, we obtained the Philadelphia REACH I dataset and posed the following research questions:

What is the relationship between caregiver well-being (PCI) and the level of care that these caregivers provide to persons with ADRD?

Can caregiver well-being (PCI) predict quality (CMI and TMSI) of care at 12 months?

If the results of the analyses show that caregiver well-being is related to the level or quality of care provided, then additional resources could be targeted toward improving caregiver well-being (for example, counseling, educational programs, skills enhancement opportunities, etc.) as a means of enhancing the level or quality of care provided to persons with ADRD.

## Methods

### Reach I

The REACH I research project (1996–2001) [17] involved six sites in the United States. The project was designed to investigate promising and innovative interventions for family caregivers of persons with ADRD or other dementias. The Philadelphia site examined the effects of the Environmental Skill-Building Program (ESP) (currently renamed and referred to as Skills_2_Care^R^) on caregiver well-being and care recipient functioning.

Caregivers who participated in REACH I had to be at least 21 years of age and they had to be providing at least 4 h of care per day for 6 months or more to a live-in loved one with ADRD. In Philadelphia, caregivers were recruited from the local area agency on aging (Philadelphia Corporation for Aging) and from media announcements. Follow-up lasted a maximum of 12 months. Detailed information about REACH I’s Philadelphia site, including participant eligibility criteria and selection methods, as well as the delivery characteristics of the intervention, have been reported elsewhere [14].

For the present analysis, we used demographic, caregiver well-being (PCI), and level of care variables from baseline. In addition, quality of care data from the 12-month follow-up were used.

### Variables

#### Main effect variable: perceived change index (PCI)

Gitlin et al. measured caregiver well-being in the form of the perceived change index (PCI). The PCI is a self-report tool to measure caregivers’ own appraisal of the levels of improvement or deterioration in their well-being [18]. Well-being is an important component of overall QoL that is closely related to health-related quality-of-life (HRQOL), a term used to distinguish aspects of QoL that are health-related from those that are not [19, 20]. HRQOL has also been described as a measure of perceived well-being [20].

The PCI was specifically developed to measure caregiver appraisals of self-improvement or decline in well-being and has since been used in other caregiver intervention trials [18]. The PCI is a 13-item instrument that uses a 5-point scale to rate whether a caregiver’s life situation has become worse (1) or improved (5) over the past month. Examples of scale items include caregivers’ ability to sleep through the night, ability to manage day-to-day caregiving, and feelings of being overwhelmed [18]. In support of its construct validity as a measure of caregiver well-being, higher PCI scores were found to be associated with fewer depressive symptoms, more activity engagement, and greater perceived benefits from caregiving [18]. Psychometric analyses suggest the PCI is valid and internally consistent (Cronbach’s alpha = 0.90) [18].

#### Outcome variables: level and quality of care provided by caregivers in ADRD

Based on the variables available in the REACH I dataset, we defined ‘level of care’ as the total number of hours per week that caregivers spent providing care for their loved ones with ADRD [13, 14]. This included the total amount of time spent helping with ADLs and IADLs. Since REACH I contained data on level of care at baseline only, we conducted a cross-sectional analysis of the association between PCI and level of care.

We used two measures from REACH I to operationalize quality of care: caregiver mastery (or “proficiency”) and task management. Caregiver mastery was measured with the Caregiving Mastery Index (CMI) from Lawton et al. [21]. The CMI is a six-item scale that uses a 5-point Likert format ranging from 1 (never) to 5 (always). A higher score means greater mastery of the caregiving role. Items on the CMI include questions such as “How often do you feel you should be doing more for the care recipient?” Regarding the psychometric properties of the CMI, the coefficient of internal consistency (a measure of the correlations between different items on the test) was found to be 0.66 in the REACH I study and 0.71 in another study of 74 caregivers that was designed to investigate caregiver appraisal of the caregiving process (e.g., caregiving satisfaction and caregiving impact) [14, 21].

Task management was measured with the task management strategy index (TMSI), a scale shown to have adequate psychometric properties. The TMSI is a 19-item scale that measures the extent to which positive caregiving strategies were used to manage ADL dependence and problem behaviours in care recipients. The TMSI also uses a 5-point Likert format from 1 (never) to 5 (always). Higher scores on the TMSI indicate greater use of such strategies. Examples of items include the extent to which caregivers employed visual and tactile cueing or short instructions to communicate with their loved ones. Regarding the psychometric properties of the CMI, the coefficient of internal consistency in REACH I was found to be 0.77 [22].

Since data on quality of care (CMI and TMSI) were available at baseline and 12-month follow-up periods, we conducted a longitudinal analysis to see if caregiver well-being can predict quality of care at 12-months.

#### Socio-demographic variables (covariates)

We examined the impact of several socio-demographic variables as covariates in all analyses. These variables included age, gender, income, education, and employment status. Research has shown that age and gender are inversely associated with well-being because women and older participants are more likely to report higher rates of disability [23, 24]. Regarding level or quality of care, older caregivers may be less able to provide the same level or quality of care as younger caregivers because of factors such as decreased mobility or increased health challenges [24]. Traditional, gender-prescribed roles might differentiate the type of care provided by male and female caregivers in certain areas such as assistance with activities of daily living.

Higher income and education are positively associated with well-being because both could generate the resources needed to provide higher levels and better quality of care than what would be the case if caregivers had lower income and education [25]. For example, caregivers with higher income could hire a substitute carer for their loved one to provide round-the-clock care, thereby leading to higher levels of care. High-income caregivers could also purchase better quality of care by hiring caregivers with specialized skills in ADRD care provision. Better-educated caregivers may be more aware of, and therefore more likely to seize, opportunities that could lead to higher levels and better quality of care for their loved ones. For example, these caregivers may be more likely to conduct research into support services such as respite care to provide better care [25].

Employment may be negatively associated with well-being because caregivers who work may experience job-related stresses (burnout, tiredness, etc.) that impact well-being [26]. Conversely, these stresses might entail less impact for caregivers who do not work. Also, employment may be negatively associated with level and quality of care because caregivers who work may be unable to devote as much time or effort to caring as would caregivers who do not work.

### Statistical analyses

We computed descriptive statistics for all variables to assess the variability of the data. We used the independent t test (continuous measures) and the Chi square test (categorical measures) to compare participants who completed 12 months of follow-up with participants who provided baseline data yet were lost to follow-up. For the outcome variables that were measured longitudinally (i.e., well-being, mastery, task management), we conducted paired samples t-tests to compare the mean values measured at baseline with the mean values measured at the 12-month follow-up time [27, 28].

Linear regression analysis was used to examine the relationships between PCI and the level or quality of care provided to persons with ADRD [29, 30]. Separate analyses were performed for level and quality as outcomes. We adjusted all regression analyses for the covariates discussed above. We also controlled for whether caregivers were in treatment (ESP intervention) or control groups. We coded categorical variables (income, employment status, and sex) into dummy variables.

We used IBM Statistics (SPSS) version 22 (IBM Corporation, Armonk, NY) and SAS version 9.2 (The SAS Institute, Cary, NC) to conduct the statistical analyses. The level of statistical significance was set at p < 0.05.

This study adheres to the Strengthening the Reporting of Observational Studies in Epidemiology (STROBE) Statement [16].

### Ethics, consent and permissions

The Philadelphia REACH data collection site obtained ethics clearance from the Thomas Jefferson University Institutional Review Board (Control number: 95.9074). All participants gave informed consent to participate.

## Results

### Caregiver sample

The Philadelphia site of REACH I initially contacted 413 caregivers, of whom 290 met the eligibility criteria for participation. Of that number, 255 caregivers agreed to participate in the study [14] and 125 (49.0 %) remained after 12 months of follow-up [31]. Reasons for drop-out included the death of the care recipient (n = 40), placing the care recipient in a long-term care facility (n = 39), missing the follow-up interview (n = 26), or withdrawing from the study (n = 25).

Table 1 presents the demographic profile of the 255 caregivers at baseline, as well as the 125 eligible participants remaining at the 12-month follow-up. At baseline, caregivers had a median age of 60 years, most (75 %) were female, and the majority (76 %) obtained at least a high school education. Most (67 %) of the caregivers were unemployed and 76 % had an annual income of less than $40,000. Although the majority of caregivers were married (58 %), most (61.2 %) were not the spouses of care recipients.

**Table 1:** Caregiver demographics

Variable	T_0_ (n = 255)	T_1_ (n = 125)
Age in years, median (25th, 75th percentile)	60 (50, 73)	59.0 (50, 70)
Gender, n (%)		
Male	65 (25)	26 (21)
Female	190 (75)	99 (79)
Educational achievement (ISCED classification), n (%)		
<High school	61 (24)	28 (23)
High school grad	84 (33)	44 (35)
>High school	110 (43)	53 (42)
Employment status, n (%)		
Full-time	60 (24)	34 (27)
Part-time	24 (9)	11 (9)
Unemployed	171 (67)	80 (64)
Income category (LICO), n (%)		
Low income	115 (46)	55 (45)
Moderate income	79 (32)	38 (31)
Middle class income	37 (15)	22 (18)
High income	16 (7)	7 (6)
Marital status, n (%)		
Never married	44 (17)	16 (13)
Married or living as married	148 (58)	70 (56)
Widowed/divorced/separated	63 (25)	39 (31)
Relation to care recipient, n (%)		
Spouse	99 (39)	43 (34)
Child	121 (47)	65 (52)
Other family	35 (14)	17 (14)
Years of caregiving, mean (SD)	4 (4)	4 (4)
Caregiving time/week in minutes, median (25th, 75th percentile)	300 (180, 480)	NA

*ISCED* Educational achievement classified based on the International Standard Classification of Education; *LICO* low income is based on the Federal Low-Income Cut-Offs of <$20,000; moderate income is $20,000–39,999; middle class income is $40,000–59,999; high income is ≥60,000; *T*_*0*_ baseline; *T*_*1*_ 12-month follow-up; *n* number of participants

Comparisons of participants at the 12-month follow-up (n = 125) and participants who were lost to follow-up between baseline and 12 months (n = 130) showed that these two sets of participants were not statistically significantly different on any of the variables examined in this study (all p values for comparisons were greater than 0.05).

### Change in PCI scores and quality of care over time

The mean PCI score increased by 0.12 (95 % confidence interval [CI] −0.23 to −0.01) between baseline and follow-up, meaning that caregiver well-being increased over time. This increase occurred because participants in the ESP intervention improved in well-being relative to those in the control group (3.00 vs 2.89; p value = 0.082), although the difference was not statistically significant [14]. The difference in mean score (−0.02) between baseline and 12-month follow-up on the CMI was not statistically significant (95 % CI −0.13 to 0.08). The difference in mean score (−0.07) on the TMSI was also not statistically significant (95 % CI −0.16 to 0.02).

### Caregiver well-being (PCI) and level or quality of care

Results of the regression analyses are presented in Table 2. The table shows the results of the cross-sectional analysis between caregiver well-being (PCI) and the amount of time (in minutes per week) caregivers spent providing care for their loved ones (level of care). The table also shows the results of the longitudinal analysis between caregiver well-being and caregiver mastery, as well as the longitudinal analysis between caregiver well-being and task management. An inverse yet statistically non-significant relationship was found between caregiver well-being and the amount of time that caregivers spent providing care for their loved ones. For every 1-unit increase in PCI score, caregivers spent an average of 4 min less per week providing care for their loved ones (95 % CI −77 to 69 min).

**Table 2:** Regression coefficients

Independent	Level of care	Quality of care (CMI)	Quality of care (TMSI)
Variables	Mean (95 % CI)	Mean (95 % CI)	Mean (95 % CI)
Well-being (PCI) (T0)	−4.00 (−76.00, 69.00)	0.10 (−0.10, 0.30)	0.20 (−0.10, 0.40)
CG age in years	−0.34 (−4.00, 3.00)	−0.00 (−0.01, 0.00)	−0.00 (−0.02, 0.00)
High income	−115.00 (−277.00, 47.00)	0.20 (−0.20, 0.60)	0.10 (−0.43, 0.60)
CG years of education	−1.00 (−19.00, 17.00)	−0.00 (−0.05, 0.04)	0.03 (−0.03, 0.10)
Full-time employment	−77.00 (−169.00, 15.00)	−0.02 (−0.30, 0.20)	−0.05 (−0.33, 0.20)
Years taken care of CR	9.00 (−1.30, 19.30)	−0.02 (−0.05, 0.00)	0.02 (−0.01, 0.05)
CG female sex	79.00 (−15.50, 173.00)	−0.21 (−0.50, 0.04)	0.10 (−0.20, 0.40)
ESP intervention	−72.74 (−148.00, 3.00)	0.11 (−0.10, 0.30)	0.10 (−0.10, 0.30)

*CG* caregiver; *CR* care recipient; *CMI* (12-months longitudinal follow-up analysis) caregiver mastery index; *ESP* intervention (received/not received); Full-time employment versus unemployed; High income versus low income = ≥60,000 versus ˂$60,000 (income categories collapsed because of multicollinearity); *Level of care* caregiving time per week in minutes (cross sectional analysis at baseline); *T0* baseline; *TMSI* (12-month longitudinal follow-up analysis) task management strategy index

Also, for every one-unit increase in caregiver well-being, the CMI score increased by an average of 0.10 points, although the association was not statistically significant (95 % CI −0.1 to 0.3). For the relationship between the PCI and TSMI, a one-unit increase in well-being led to an average 0.20 increase in the TMSI, which was also not statistically significant (95 % CI −0.1 to 0.5).

### Effect of caregiver socio-demographic characteristics

None of the socio-demographic variables tested were statistically significant in the regression models (Table 2). Also, caregivers who received the ESP did not provide significantly better level (amount of time) or quality of care (CMI and TMSI) compared to caregivers who did not receive the intervention.

## Discussion

We examined the relation between caregiver well-being and the level and quality of care provided to persons with ADRD, controlling for important socio-demographic variables such as caregiver age, employment status, income, marital status, and sex and for 12 month analyses, group allocation. The socio-demographic profile (e.g., proportion of males/females, age distribution, marital status, etc.) of the ADRD caregivers enrolled in the Philadelphia REACH I study was similar to that of caregivers in other sites of the REACH I study [17]. For example, the average age for caregivers at the six intervention sites of the REACH study was 62 years compared to 60 years for the Philadelphia site [17]. Other studies examining caregivers in North America have also reported similar demographic data, including the proportion of females, income levels, and marital status [4, 8, 9].

Despite an increase in caregiver well-being scores over the course of follow-up, mastery and task management strategy scores did not exhibit statistically significant changes during this time (e.g., the intervention or time did not affect these outcomes). Regression analyses showed that at 12 months, the PCI was not associated with mastery or task management strategy, controlling for various socio-demographic variables, and receipt of ESP.

Overall, the REACH I dataset provided an opportunity to explore an important, but unexplored area, and we did not find any statistically significant associations. The longitudinal nature of the data made it possible to examine changes in caregiver well-being and quality of care over a 12-month period. Also, several important covariates were examined in this study. However, there were several limitations with the REACH I dataset that prevented a full evaluation of the research question. We could not conduct a longitudinal analysis of “caregiver time” because only baseline data were available for this variable. The potential for reverse causality in the assessment of cross-sectional associations suggests that the inverse relationship observed between the PCI score and caregiver time could mean that lower levels of caregiver time lead to higher feelings of perceived well-being. Another limitation of the study was that the REACH I data included only those participants who provided at least 4 hours of care per day. This made it impossible to examine how the well-being of caregivers providing less than 4 hours of care per day affect the level or quality of care provided.

We also note that the sample size at baseline (255 caregivers) was relatively small and there was a high rate of attrition over time with 130 (51.0 %) caregivers dropping out over 12 months. This affected the power of the study. Assuming a level of significance of 0.05 and a power of 0.8, a post hoc power analysis showed that between 1009 and 19,133 participants would be needed to detect the difference of 0.02 on the CMI, depending on the correlation between CMI scores at baseline and follow-up. For the TMSI, between 125 and 2329 participants would be needed to detect the difference of 0.07, depending on the correlation.

In addition, the REACH I database did not contain data to allow us to properly control for the severity of illness of the care recipients. Severity of illness is an important predictor of the manner in which ADRD manifests itself in care recipients [32]. As the disease progresses, care recipients become more likely to exhibit troublesome behaviours and they become more reliant on caregivers for help with instrumental and basic ADLs. Therefore, disease severity may have deleterious effects on caregiver well-being and the level or quality of care provided.

The limitations of the available data suggest that further research is required to examine the relationship between caregiver well-being and the level or quality of care provided to persons with ADRD. Further research would ideally require a longitudinal study to directly investigate the association between caregiver well-being and the level or quality of care over a reasonable follow-up time. One potential length of follow-up would be 3–5 years, which has been estimated to be the mean survival time for persons diagnosed with ADRD [33]. Further research would also require appropriate instruments for measuring level of care and quality of care. Any such study would have to enrol an adequate number of participants to have sufficient power to detect statistically significant associations between caregiver QoL and the level or quality of care provided.

Regarding sample size for the proposed study, a reliable linear regression model is obtained when the ratio of study participants to variables in the full model falls between 10 and 20 [34]. In the proposed longitudinal study, it is estimated that the maximum number of variables will be 30, including dummy variables for interview questions with categorical responses (e.g., male and female). Each level of a categorical variable counts as one variable in this formulation. Other variables would include socio-demographic variables like age, income (low versus high), employment status (unemployed, part-time, and full-time), and severity of illness (moderate versus severe). Given the estimate of 30 variables in total, at least 300 participants will be needed to build a reliable regression model. A reliable model is one where the regression coefficients are stable, such that if the model were run on multiple independent datasets, then the resulting coefficients would be similar to one another across datasets [34].

Research to study the link between caregiver well-being and the level or quality of care provided to persons with ADRD is important because the results could lead to interventions that help caregivers provide better care. Examples of interventions include the development of a practical skills list (for example, stress management techniques) that would be taught to caregivers to improve their quality-of-life. Further research could also provide valuable insights into the factors that influence *how* caregivers fulfill their caregiving role. Aspects of well-being that are found to be more closely associated with the level or quality of care provided could be specifically targeted for improvements.

## Conclusions

The findings from this study did not show a statistically significant association between caregiver well-being and the level and quality of care provided to persons with ADRD. Further research is required to investigate whether caregiver well-being (or QoL in general) is associated with the level or quality of care provided.

## Abbreviations

AD: alzheimer’s disease
ADL: activities of daily living
ADRD: alzheimer’s disease and related disorders
CG: caregiver
CR: care recipient
CI: confidence interval
CMI: caregiver mastery index
ESP: environmental skill-building program
HRQOL: health-related quality-of-life
IADLs: instrumental activities of daily living
n: number of participants
NIH: National Institutes of Health
ISCED: International Standard Classification of Education
LICO: low-income cut-offs
PCI: perceived change index
QoL: quality-of-life
REACH I: resources for enhancing alzheimer’s caregiver health
REB: Research Ethics Board
STROBE: strengthening the reporting of observational studies in epidemiology
T_0_: baseline
T_1_: 12-month follow-up
TMSI: task management strategy index
